# Biophysics, pathophysiology, and pharmacology of ion channel gating pores

**DOI:** 10.3389/fphar.2014.00053

**Published:** 2014-04-03

**Authors:** Adrien Moreau, Pascal Gosselin-Badaroudine, Mohamed Chahine

**Affiliations:** ^1^Centre de Recherche de L'Institut Universitaire en Santé Mentale de QuébecQuebec City, QC, Canada; ^2^Department of Medicine, Université LavalQuebec City, QC, Canada

**Keywords:** ion channels, voltage sensor domain, channelopathies, gating charge transfer center, sodium channels, omega pores

## Abstract

Voltage sensor domains (VSDs) are a feature of voltage gated ion channels (VGICs) and voltage sensitive proteins. They are composed of four transmembrane (TM) segments (S1–S4). Currents leaking through VSDs are called omega or gating pore currents. Gating pores are caused by mutations of the highly conserved positively charged amino acids in the S4 segment that disrupt interactions between the S4 segment and the gating charge transfer center (GCTC). The GCTC separates the intracellular and extracellular water crevices. The disruption of S4–GCTC interactions allows these crevices to communicate and create a fast activating and non-inactivating alternative cation-selective permeation pathway of low conductance, or a gating pore. Gating pore currents have recently been shown to cause periodic paralysis phenotypes. There is also increasing evidence that gating pores are linked to several other familial diseases. For example, gating pores in Na_v_1.5 and K_v_7.2 channels may underlie mixed arrhythmias associated with dilated cardiomyopathy (DCM) phenotypes and peripheral nerve hyperexcitability (PNH), respectively. There is little evidence for the existence of gating pore blockers. Moreover, it is known that a number of toxins bind to the VSD of a specific domain of Na^+^ channels. These toxins may thus modulate gating pore currents. This focus on the VSD motif opens up a new area of research centered on developing molecules to treat a number of cell excitability disorders such as epilepsy, cardiac arrhythmias, and pain. The purpose of the present review is to summarize existing knowledge of the pathophysiology, biophysics, and pharmacology of gating pore currents and to serve as a guide for future studies aimed at improving our understanding of gating pores and their pathophysiological roles.

## Introduction

**Figure 1:**
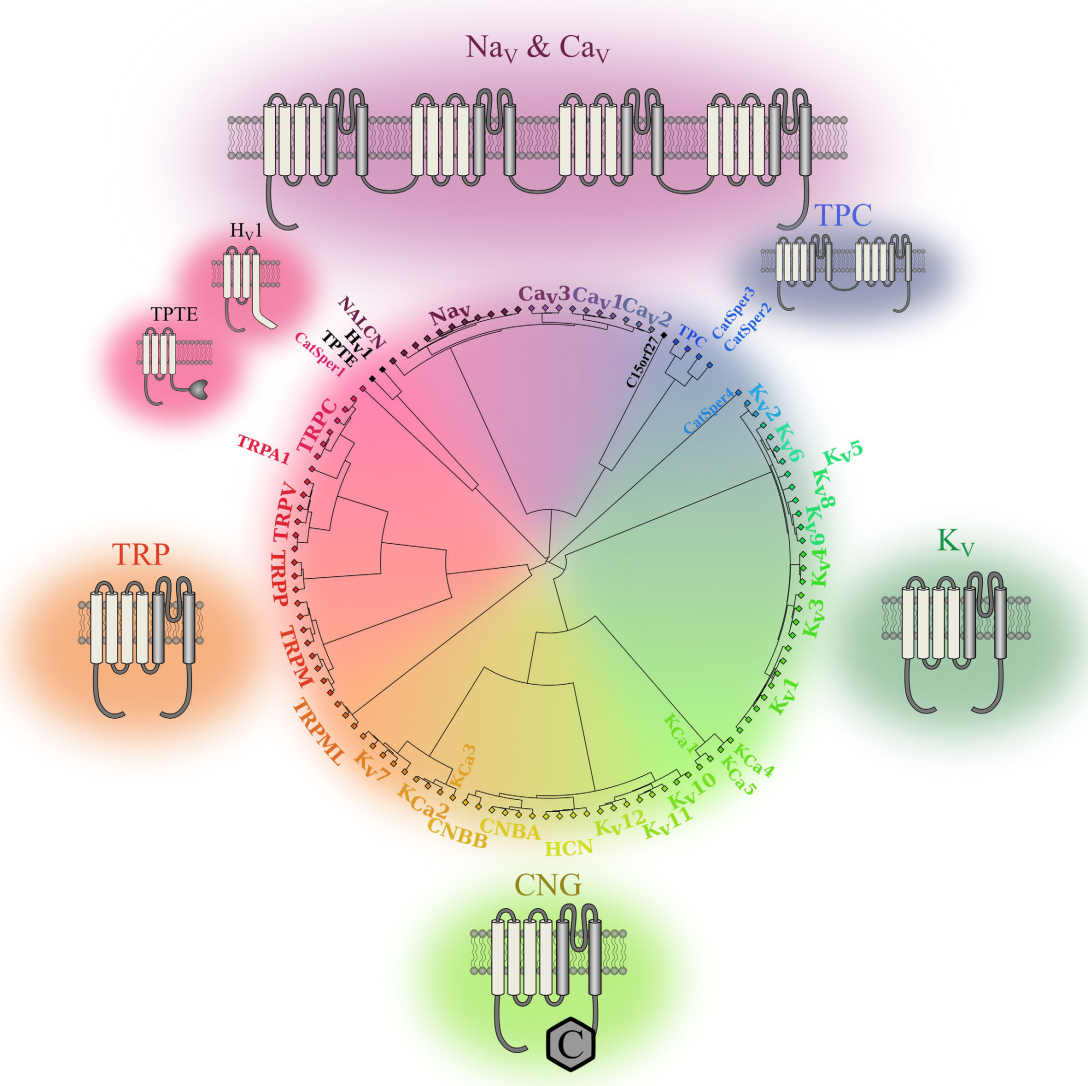
**Phylogenic representation of the VSD-featuring protein superfamily**. The relationships between the amino acid sequences of various human proteins that feature one or more VSD motifs are represented as a circular phylogenic tree. The proteins are from the VGIC family (Yu and Catterall, 2004). Channels that do not feature a VSD motif were eliminated while proteins known to feature a VSD motif were included (Iwasaki et al., 2008; Musset et al., 2011). The complete sequences for each protein were aligned using Clustal Omega (Sievers et al., 2011). The phylogenic tree was then created using a custom Matlab script using the single linkage method. It highlights the five subgroups of the human VSD-featuring protein superfamily. Na_v_ and Ca_v_ channels are in shades of purple. These proteins have a VSD motif in each of their four homologous domains. TPCs are in blue. The fact that they feature a VSD motif in their two homologous domains sets them apart from the other members of the VSD-featuring protein superfamily. The structures of K_v_ channels are in dark green. Since some of these channels share structural similarities with calcium-activated potassium channels (K_Ca_) channels and CNG channels, the K_v_7, K_v_10, K_v_11, and K_v_12 channels were placed next to the CNG and K_Ca_ families. The structures of the CNG and K_Ca_ families are in light green, while the structures of TRP channels are in orange. Members of the PD-lacking group of proteins are in black. The 2-D structures show the VSDs in light gray and the PDs in dark gray.

In the late 1940s, Hodgkin and Huxley were the first to highlight the importance of ionic movements in cell excitability. They showed that the process was mediated by dedicated structures now known as ion channels (Hodgkin and Huxley, 1952). Thirty-two years later, the first voltage sensitive ion channel was cloned (Noda et al., 1984). To date, at least 140 similar structures have been identified and assigned to the voltage gated ion channel (VGIC) superfamily (Yu and Catterall, 2004). Most of these VGICs (113 of 140) feature a voltage sensor domain (VSD) and thus belong to the VSD-featuring protein superfamily (Figure 1). Two structures are very common in this superfamily: the pore domain (PD) and the VSD. Based on ion selectivity, functional similarities, and structural homology, this superfamily can be divided into five main types: proteins lacking a PD, voltage gated sodium and calcium channels (Na_v_, Ca_v_), voltage gated potassium channels (K_v_), cyclic nucleotide gated channels (CNG), and transient receptors potential channels (TRPs).

The PD motif is present in channels that require the assembly of four VSD and PD motifs to form functional units. The PD is composed of two transmembrane (TM) segments and a re-entrant pore loop that links the segments (Figure 1). The assembly of four independent motifs forms tetrameric units that are frequently found in K_v_, CNG, and bacterial Na_v_ channels. The assembly of four motifs into four domains of the same protein (DI to DIV) is a characteristic of mammalian Na_v_ and Ca_v_ channels (Figure 1). The two-pore channels (TPC) sub-family should be treated as an exception since it requires the assembly of only two units to form a functional unit. Each unit features two domains that assemble as a dimer to create a functional channel. The assembly of four PD motifs creates a permeation pathway that is responsible for the passage of ions from one side of the membrane to the other. The ionic selectivity of the channels is mainly provided by four to five highly conserved amino acids in the PD of Na_v_, Ca_v_, and K_v_ channels [DEKA, EEDD, and (T/S)XG(Y/F)G, respectively] (Heinemann et al., 1992; Doyle et al., 1998; Valiyaveetil et al., 2006).

VSDs are composed of four TM segments (S1–S4) (Figure 1) and are present in most VGICs and in a few other proteins such as the voltage gated proton channel (H_v_1) and TPTE. These proteins do not require the assembly of four domains to create a functional unit. To date, only the H_v_1 and TPTE proteins have been assigned to the category of proteins that do not feature a PD motif. However, the *C15orf27* protein, which may contain a VSD, could likely be added to this category (Musset et al., 2011). Even if this category of proteins does not contain many members, studying it may lead to the discovery of similar proteins that play important physiological roles (Capasso et al., 2011).

**Figure 2:**
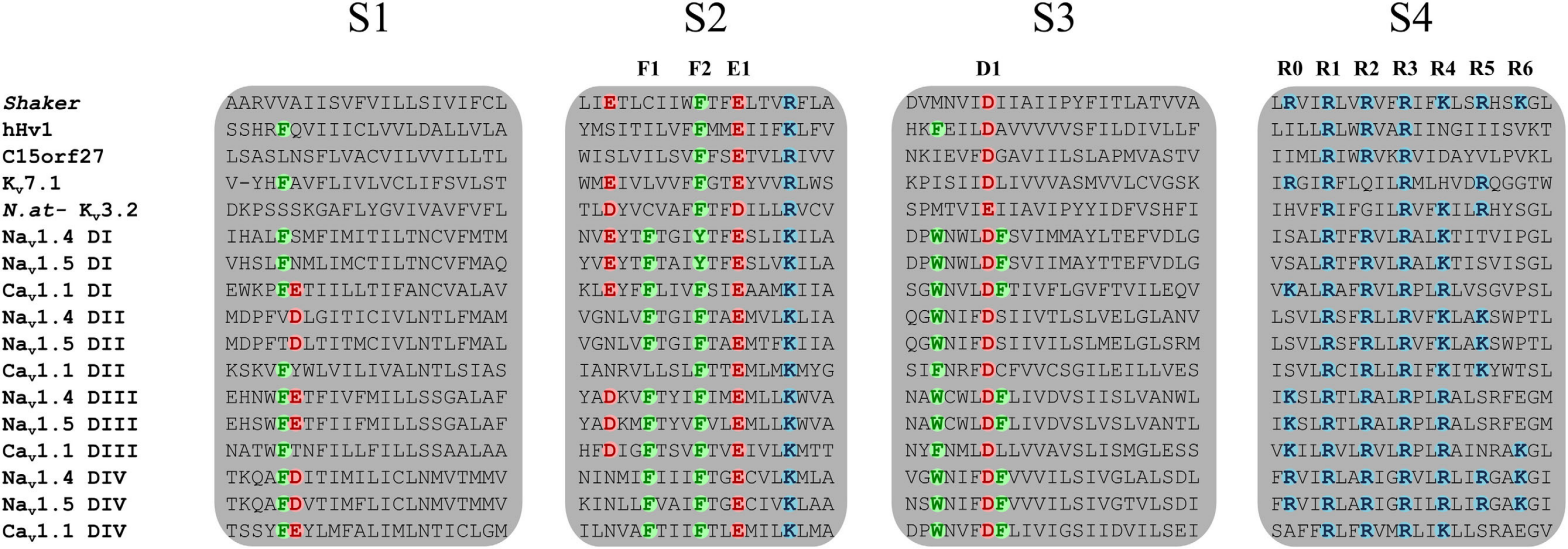
**Sequence alignment of the four TM segments of VSDs from the VSD-featuring protein superfamily**. The Na_v_1.4, Na_v_1.5, Ca_v_1.1, and K_v_7.1 channels are shown. Members of the VSD-featuring protein superfamily such as H_v_1 and *C15orf27* are also shown. The S1, S2, and S3 segments contain highly conserved negatively charged and aromatic residues while the S4 segment contains at least three positively charged residues. Highly conserved residues are in bold colors. Positively charged residues are in blue (arginine or lysine), negatively charged residues are in red (aspartate or glutamate), and aromatic residues are in green (phenylalanine or tyrosine). The residues involved in the GCTC are annotated as F1, F2, E1, D1, and R0 to R6.

The voltage sensitivity of the VSD-featuring channel and protein superfamily is provided by the interaction of several highly conserved charged residues. The S4 segment contains at least three positively charged residues (arginines and lysines) (Figure 2) while the S1, S2, and S3 segments contain organized structures of highly conserved negatively charged amino acids (aspartate and glutamate) as well as highly conserved aromatic amino acids (tryptophan, phenylalanine, or tyrosine) (Figure 2). Under the effect of voltage, the S4 segment moves toward the extracellular medium and causes a conformational change that results in the opening of the pore (Yang and Horn, 1995; Yang et al., 1996). The nature of this movement has been the subject of much debate. Three main models had been proposed: the sliding helix model (Catterall, 1986), the helical-screw model (Guy and Seetharamulu, 1986) and the paddle model (Jiang et al., 2003). Recent progress in modeling techniques has shown that the displacement of S4 is likely a blend of all three models (Delemotte et al., 2011). However, in order to clarify and better understand the nature of the movement of S4, studies have focused on the highly conserved residues shown in Figure 2. In 2004, Starace and Bezanilla substituted the first highly conserved arginine by a histidine on the S4 segment of the *Shaker* K^+^ channel (R362H) (Starace and Bezanilla, 2004). This mutation was initially used to study the extracellular accessibility of the residue at rest. Interestingly, this study revealed that a proton (H^+^) leak current was created following the arginine-to-histidine substitution (Starace and Bezanilla, 2004). The authors concluded that the paddle model would have to be modified to reconcile the proposed structure and the experimental data. In a subsequent study, Tombola et al. showed that the neutralization of the first arginine on the S4 segment induced a cation leak through the VSD (R362A/C/H/S/V in the *Shaker* channel). The newly formed pore through which this leak occurred was called an omega pore (also known as a gating pore) to differentiate it from the physiological permeation pathway known as the alpha pore (Tombola et al., 2005).

## The voltage sensor domain and the structure of gating pores

**Figure 3:**
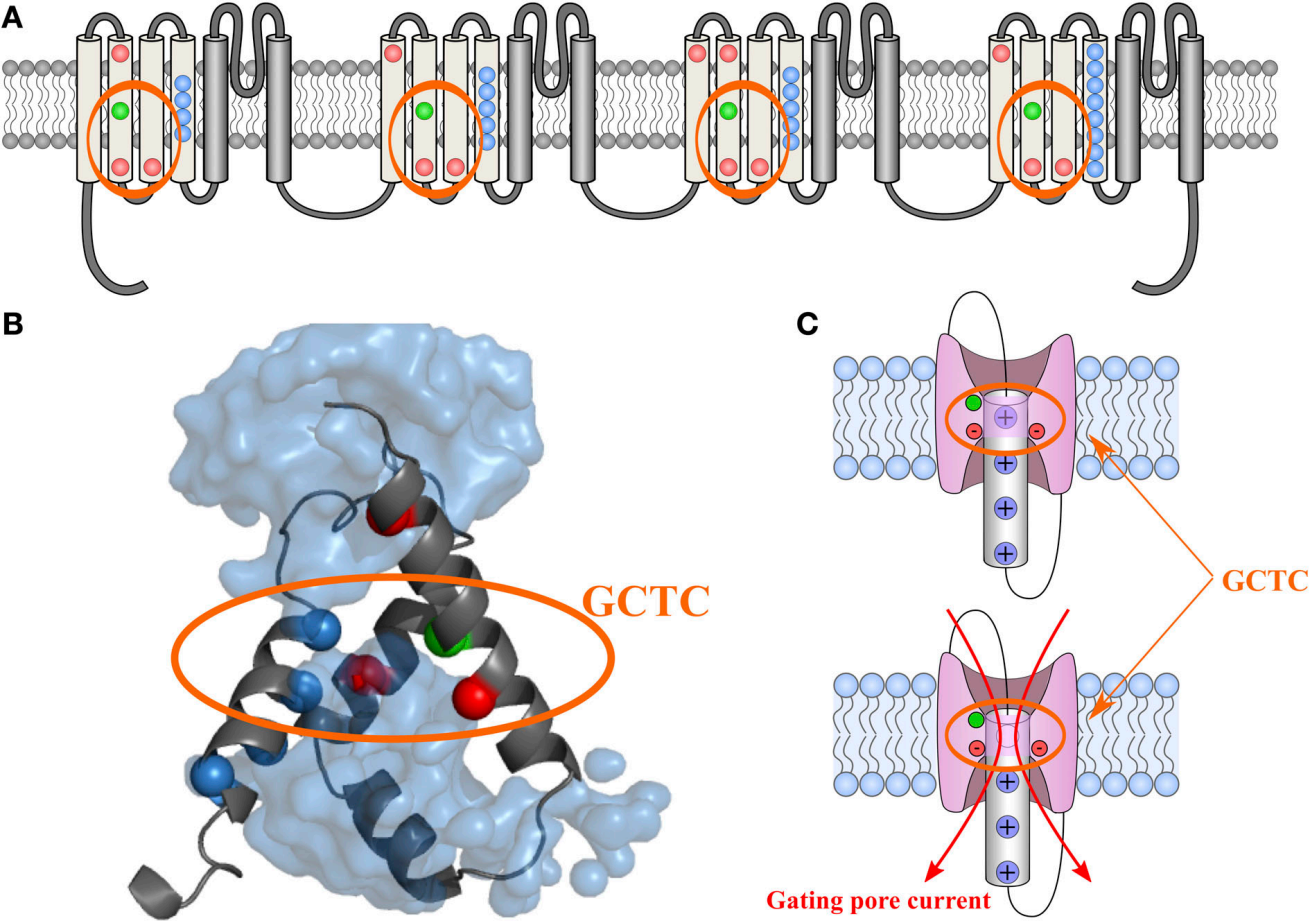
**2-D and 3-D schematic representations of the GCTC**. The 2-D structure of the Na_v_1.4 channel is shown in **(A)**. The VSD motifs of the four domains are in light gray while the PD motifs are in dark gray. Highly conserved amino acids in the VSD motifs are in color. The blue, red, and green dots represent positively charged, negatively charged, and aromatic residues, respectively. The 3-D assembly of the Na_v_1.4 DI VSD motif in its resting state, as published by Gosselin-Badaroudine et al. is shown in **(B)** (Gosselin-Badaroudine et al., 2012a). The model features the water crevices (light blue), the conserved arginine and lysine residues (dark blue), the conserved aspartate and glutamate residues (red), and the conserved tyrosine residue (green). As can be seen from the model, the first arginine residue is close to the GCTC. A schematic representation of the VSD motif in its resting state in the presence and absence of the first positively charged amino acid (the first arginine residue) is shown in **(C)**. This representation highlights the fact that the neutralization of the amino acid in the R1 position would allow the water crevices to communicate when the VSDs are in the resting state and thus induce gating pore currents (Gosselin-Badaroudine et al., 2012a).

**Figure 4:**
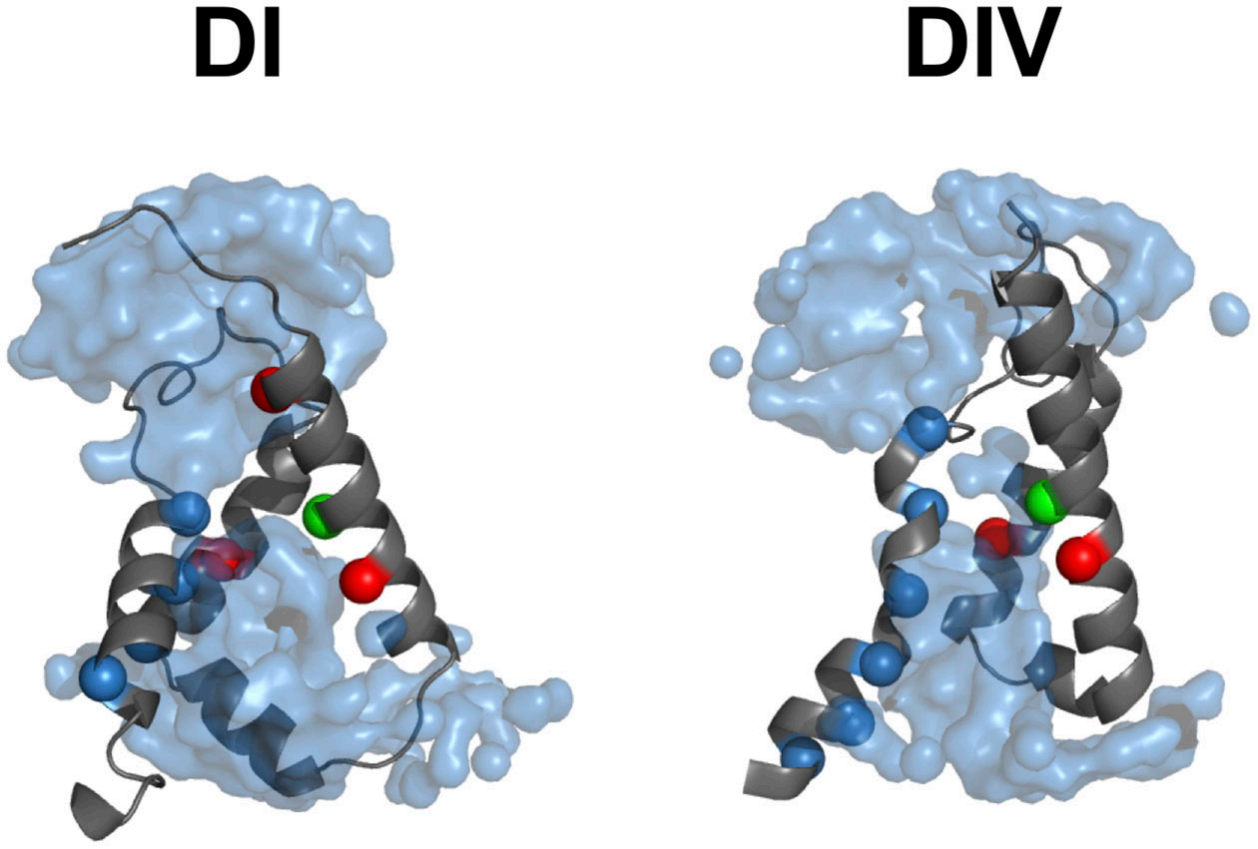
**The hydrophobic septum of domain IV is larger**. The 3-D assembly of the Na_v_1.4 DI and DIV VSD motifs in their resting state, as published by Gosselin-Badaroudine et al. (2012a). The segments S1 of both VSDs have been removed for clarity. As illustrated, the model indicates a considerably larger septum in domain IV, probably explaining its slower kinetics. Depicted here are: the water crevices (light blue), the conserved arginine and lysine residues (dark blue), the conserved aspartate and glutamate residues (red), and the conserved tyrosine residue (green).

Voltage sensitivity is important for a wide variety of physiological functions (Yu et al., 2005). Many voltage sensitive proteins (VSPs) share a common structural motif called the VSD. VSDs are assemblies of four TM segments (S1–S4) (Figure 1). The S4 segment seems to be the *central pillar* of gating pores given that it drives conformational changes of VSDs. S4 is especially affected by voltage changes since it features several highly conserved positively charged amino acids (arginines or lysines) (Figure 2). Conserved residues on S1, S2, and S3 (Figure 2) appear to be involved either in the stabilization of S4 in different conformational states (Tao et al., 2010; Pless et al., 2011) or in the shape of the water crevices surrounding S4 (Pless et al., 2011). These water crevices are very important for focusing the electric field around the S4 segment. Alterations to the water crevices may change the movement kinetics of VSDs without necessarily changing the stability of their various states (Pless et al., 2011). There is also a complex of three highly conserved amino acids at the center of each VSD. This complex forms the gating charge transfer center (GCTC) (Figure 3) (Tao et al., 2010) and is composed of one aromatic residue (phenylalanine, tryptophan, or tyrosine) and two negatively charged residues (aspartic acid and glutamic acid) (Figure 3). The aromatic residue can interact with arginines through a cation-π interaction (Pless et al., 2011), but it preferentially interacts with lysine to stabilize the activated or resting state of VSDs (Tao et al., 2010; Pless et al., 2011). The GCTC separates the intracellular and extracellular water crevices (Figure 3), which gives rise to a hydrophobic septum that also modulates the kinetics of the S4 segment. The larger the septum, the higher the free energy barrier is. As such, charged amino acids such as arginine and lysine need more energy to move through the septum. A large hydrophobic septum would delay the onset of the movement of S4 and changes its voltage sensitivity. Differences in the sizes of the hydrophobic septa of the domains of the Na_v_1.4 channel have been reported (Gosselin-Badaroudine et al., 2012a). Interestingly, the hydrophobic septum in domain IV is larger than the septa of the other domains. This is illustrated by comparing the hydrophobic regions of domains I and IV (Figure 4). This larger septum in domain IV would explain the delayed activation of this VSD (Chanda and Bezanilla, 2002). A large septum would likely involve slower conformational changes to VSDs and be linked to more stable resting states.

Neutralizing the positively charged amino acid interacting with the GCTC at a given state creates a gating pore (Starace and Bezanilla, 2004; Tombola et al., 2005). If the charges of the GCTC are not counterbalanced by the charges of S4, the region becomes hydrophilic. The change from hydrophobic to hydrophilic implies that the water crevices are no longer separated, which allows ions to permeate through the newly formed narrow pore (Figure 3). Such pores have been used to probe the structures of the VSDs of various proteins (Starace and Bezanilla, 2004; Tombola et al., 2005; Gamal El-Din et al., 2010). They have also been used to explain features of VSDs in physiological conditions (Klassen et al., 2008; Berger and Isacoff, 2011; Musset et al., 2011) as well as pathological phenotypes (Sokolov et al., 2007; Struyk et al., 2008; Gosselin-Badaroudine et al., 2012b). However, caution is warranted when drawing conclusions based solely on the existence and modulation of a gating pore current. Specifically, it has been suggested that R362 (the first arginine residue on S4) interacts with E283 (the first highly conserved glutamate on S2) in the *Shaker* channel since an E283D mutation introduced into an R362C background increases the gating pore current (Tombola et al., 2005). It appears that, in the wild type (WT) channel, R362 is closer to F290 (the first highly conserved phenylalanine on S2) (Figure 2) than E283. Residues located below this site preferentially form Zn^2+^ bridges with residues located above R362 (Lin et al., 2011). For instance, Lin et al. substituted a poorly conserved residue located below E283 (the I287 residue) with a histidine. In the background of this mutation, ~56% of the channels that also featured a R362H mutation could be momentarily trapped in their resting state when Zn^2+^ was present in the extracellular solution, indicating that Zn^2+^ can interact with both newly introduced histidine residues to form a Zn^2+^ bridge. However, in the background of the I287H mutation, the A359H substitution was more efficient at trapping the channels in their resting state since all the channels formed a Zn^2+^ bridge, indicating that I287 is closer to A359 than to R362, which would thus be located in close proximity to residues below I287. This is in agreement with reports that the positively charged residues of the S4 segment interact with the highly conserved aromatic amino acid in the S2 segment as observed in K_v_ channels (Tao et al., 2010). However, this does not negate the fact that the R362C/E283D double mutant yields a larger gating pore than the R362C single mutant, which would indicate that mutant VSDs may feature stable conformational states slightly different from their WT counterparts. While we believe in the validity of the published reports, we advise caution. Indeed, to develop an artifact-free structural model of VSDs, interactions between highly conserved amino acids uncovered by the detection of gating pore currents should be confirmed using other techniques. For example, the creation of Zn^2+^, Mg^2+^, or cysteine bridges created using less critical, more poorly conserved residues located near the interaction of interest should confirm whether or not the more poorly conserved residues are in close proximity. It would be reasonable to conclude that the interactions of conserved residues also occur in WT channels if mutant channels featuring disruptive mutations (mutations yielding gating pore currents) and less disruptive mutations (mutations yielding bridges) point to the same conclusion.

## Biophysical properties of gating pores

### Gating of gating pores

#### Voltage-dependence

**Figure 5:**
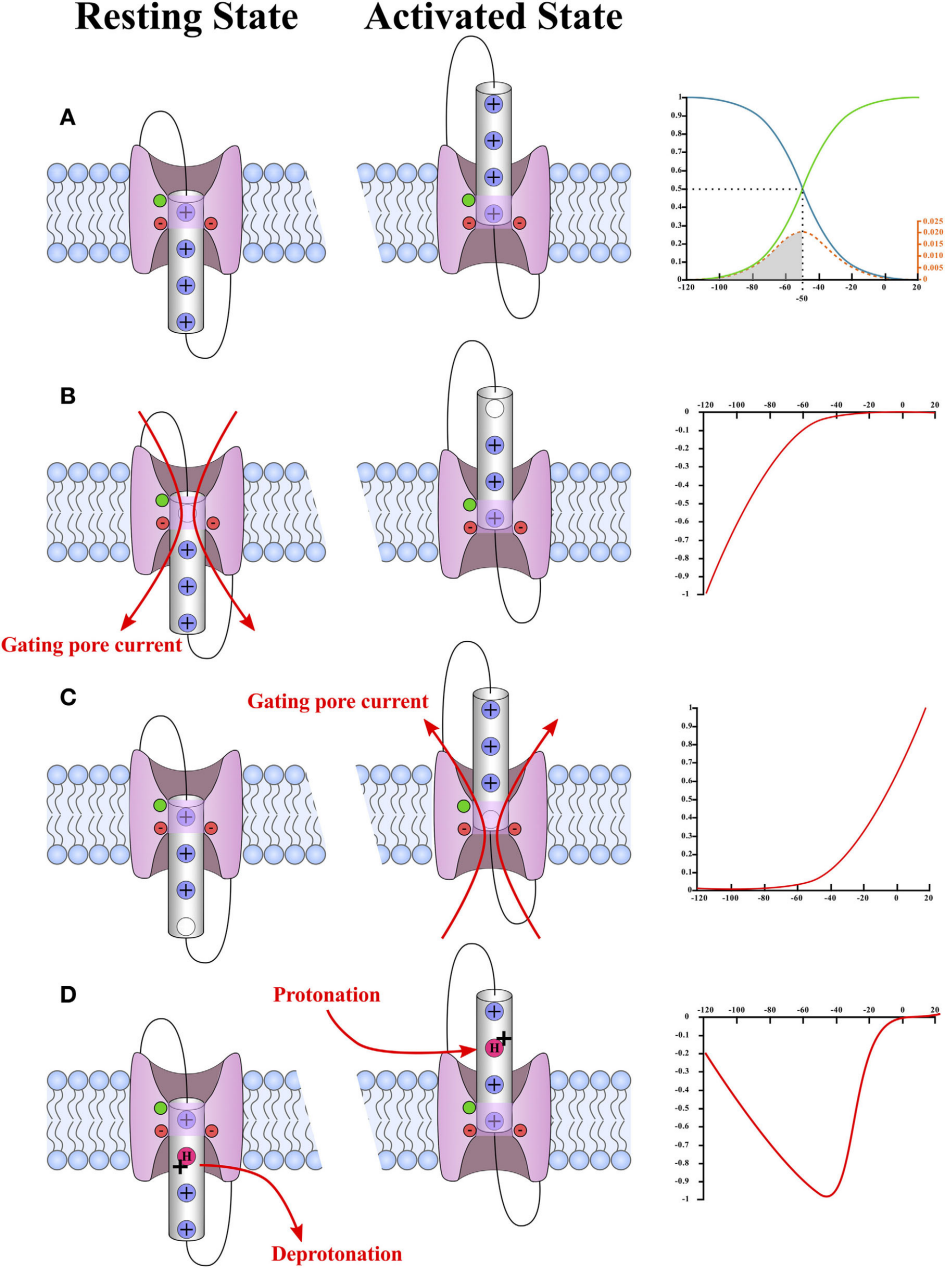
**Schematic representation of the different types of gating pores and the typical current–voltage curves of their gating pore currents**. A plot of the probability of observing a hypothetical VSD in its activated (green) or resting state (blue) is shown in **(A)**. In this case, the hypothetical VSD would have a V_1/2_ of −50 mV and a k of −12 mV. The probability as a function of voltage was calculated using Equation (2) (see text). The probability that the VSD transits from one state to another as a function of voltage is given by Equation (4) (see text) and is shown in orange. The schematic representations of the WT VSD in the activated and resting states are shown in **(A)**. The typical current–voltage curves of gating pores opened in the resting state of the VSD and in the activated state of the VSD are shown in **(B,C)**, respectively. The schematic representations of VSDs with gating pores are shown on the side. These representations highlight the fact that gating pore currents occur when the interaction between the positively charged S4 segment and the GCTC is suppressed. The highly conserved aromatic and negatively charged amino acids that make up the GCTC are shown in green and red, respectively. The schematic representations also show that gating pore currents are blocked when the S4-GCTC interaction is restored. A current–voltage curve of proton transport through the VSD as reported by Starace and Bezanilla (2001) is shown in **(D)**. The mechanism by which this transport occurs is shown on the left. Briefly, proton transport requires an arginine-to-histidine substitution. When this substitution occurs on arginine residues that interact with the GCTC when the VSD is not fully activated, no gating pore is formed in the stable states. However, the substituted residue is accessible to water in both the resting and activated states. This means that the histidine can be protonated in the activated state of the VSD and deprotonated in the resting state of the VSD.

Gating is paramount when describing ionic conductances. This property makes it possible to discriminate between ionic conductances. As mentioned previously, gating pores are open when the interaction between the S4 segment and the GCTC is abolished (Figures 3, 4), which means that gating pores can be created in both the resting state (Figure 5B) (Starace and Bezanilla, 2004; Tombola et al., 2005) and the activated state (Figure 5C) (Sokolov et al., 2008). In addition, few investigators have reported that mutations of the arginines interacting with the GCTC during the transition from the resting state to the activated state result in proton transport through VSDs, which is very similar to gating pore currents (Figure 5D) (Starace and Bezanilla, 2001, 2004).

Since the gating of gating pores is state-dependent, the probability of a gating pore being open at a given voltage is directly related to the probability of observing the VSD in the state of interest. A two-state Boltzmann distribution can be used to determine the probability of finding a VSD in a given state as a function of the membrane potential (Armstrong and Bezanilla, 1973). This allows the charge movement to be traced as a function of the voltage relationship (Q-V) (Figure 5A).

According to Boltzmann's law of distribution, the probability of observing a given particle (a VSD) in a given energy state is proportional to the following exponential (Equation 1):

(1) $$p\propto e^{-E/kT}$$

where “*k*” is Boltzmann's constant, “*T*” is the temperature, and “*E*” is the energy at which the particle is observed.

Since we assume a two-state system, the probability of observing the VSD in its activated state or in its resting state is 1. Thus, the proportion of VSDs observed in a given state is represented by Equation (2):

(2) $$P_{1}=\frac{1}{1+e^{-(E_{1}-E_{2})/kT}}$$

Since the energy is normally applied as an electrical potential, the equation is usually written as:

(3) $$P_{activated}=\frac{1}{1+e^{zq_{e}(v_{1/2}-v_{m})/kT}}$$

where “*z*” is the equivalent charge movement, “*q*_*e*_” is the elementary charge, “*V*_*m*_” is the electrical potential at which VSDs are observed, and “*V*_1/2_” is the electrical potential at which half the VSDs are in their activated state.

Such a statistical distribution implies that the VSDs transition to the activated state as a function of voltage, expressed by the following equation:

(4) $$\frac{dP_{activated}}{dV}=\frac{zq_{e}e^{(v_{1/2}-v_{m})/kT}}{kT{\left(e^{zq_{e}(v_{1/2}-v_{m})/kT}+1\right)}^{2}}$$

This indicates that not all VSDs change states at the same voltage. Moreover, the area under the energy distribution curve from 0 to a given voltage represents the proportion of VSDs that have a sufficient amount of energy to be observed in their activated state at the given voltage (Figure 5A).

The Q-V curve directly fits the Boltzmann equation of a two-state system mentioned earlier. The opening probability of the gating pore is given by the Q-V curve for VSDs for which the S4-GCTC interaction is suppressed in the activated state (Equation 3). In the case of an S4-GCTC interaction suppressed in the resting state, the opening probability is given by 1 minus the Q-V curve. This means that the gating pore can either be defined as an inward rectifying conductance if the S4-GCTC interaction is suppressed in the resting state or as an outward rectifying conductance if the S4-GCTC interaction is suppressed in the activated state (Figure 5).

H^+^ transport due to the suppression of the S4-GCTC interaction in transition states is a special case as this H^+^ transport is maximal when the transition rate of the VSD is maximal (Figure 5D). The transition rates are maximal at the V_1/2_ of the Q-V curve, which is also the voltage value at which the number of VSDs changing states is the highest (as seen by the energy distribution curve) (Figure 5). Such H^+^ transport has already been observed with *Shaker* K^+^ channels (Starace and Bezanilla, 2004).

#### Kinetics

The activation kinetics of gating pores are very rapid, with its onset being less than 1 ms. Once activated, gating pores do not inactivate since VSDs tend to stay in their activated state until the TM voltage returns to hyperpolarizing values.

Due to their nature, the biophysical characteristics of gating pores are intimately linked to the state, environment, and movement of the S4 segment. This provides several advantages, notably for structure-function studies. As such, the voltage-dependence of gating pore currents can be used as a read-out of the movement of individual voltage sensors (Gosselin-Badaroudine et al., 2012a).

### Selectivity and conductance of gating pores

#### Selectivity

**Figure 6:**
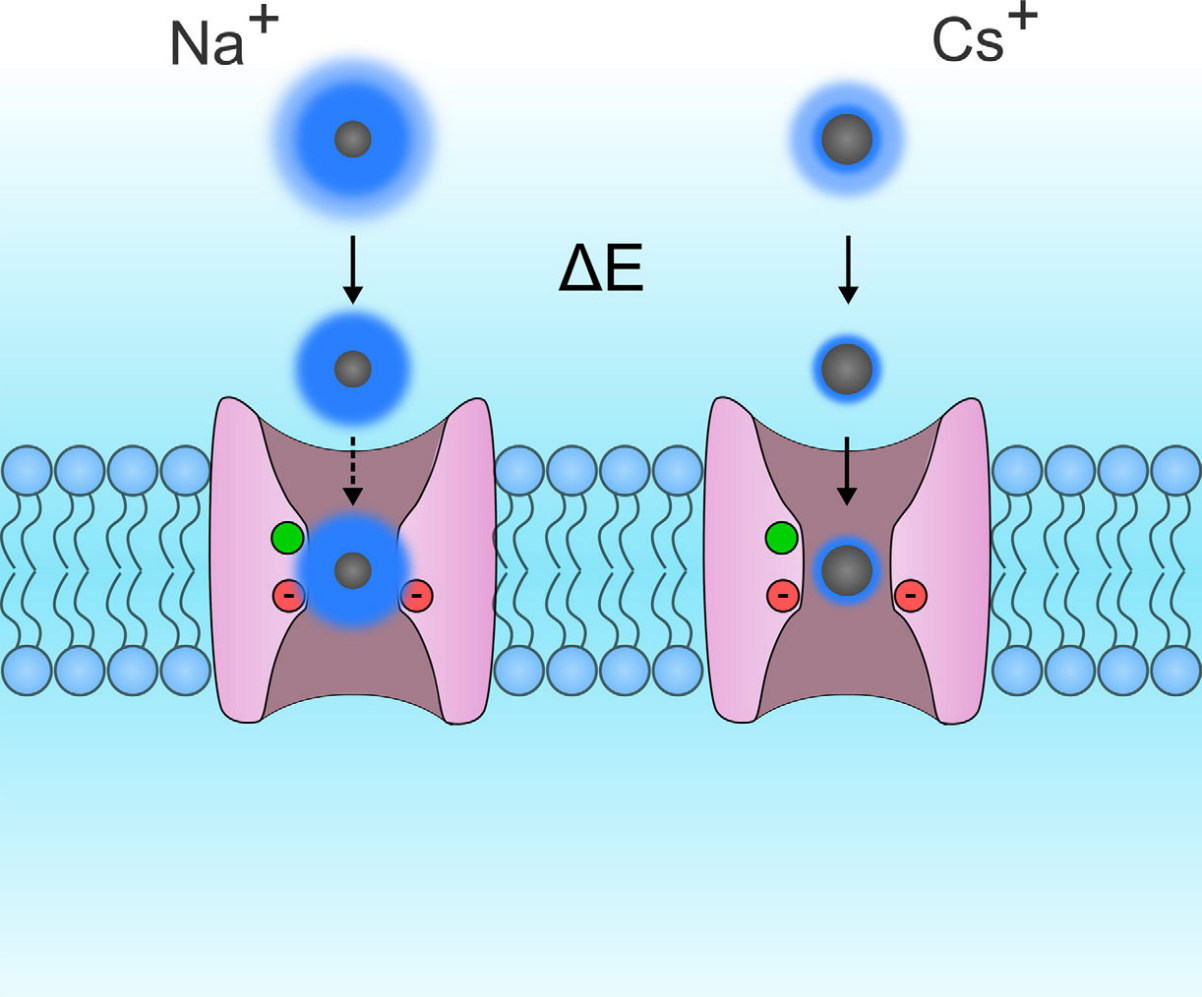
**Schematic representation of Eisenmann's principle**. This figure shows two VSDs embedded in the cytoplasmic membrane. For the sake of simplicity, the S4 segments were removed so that the permeation pathway of the gating pore could be seen more clearly. The permeation of Na^+^ and Cs^+^ ions are shown on the left and right, respectively. Ions are in gray while their hydration shells are in dark blue. The hydration shells for small monovalent ions such as Na^+^ are larger than the hydration shells for larger monovalent ions such as Rb^+^ and Cs^+^. This is due to the fact that the charge density of Na^+^ ions is larger than that of Cs^+^ ions. While both ions possess the same charge, Na^+^ ions are smaller. Na^+^ ions are more tightly bonded to water molecules and require more energy to break the bond. This energy may be provided by a binding site in the pore. In gating pores, the GCTC, which is composed of one aromatic and two negatively charged amino acids (in green and red, respectively) may act as binding sites. In such narrow pores, a weak binding site would result in a lower relative conductance of Na^+^ ions than of Cs^+^ ions. Based on currently available selectivity sequences (Table 1), gating pores would have a weak binding site.

Few studies have investigated the selectivity of gating pores (Table 1) (Tombola et al., 2005; Sokolov et al., 2007; Klassen et al., 2008; Berger and Isacoff, 2011; Francis et al., 2011). The selectivity sequences reported by Tombola et al., Sokolov et al., and Francis et al. indicate that gating pores may typically be more permeable to large cations than small cations (Tombola et al., 2005; Sokolov et al., 2007; Francis et al., 2011). The selectivity sequences thus seem to converge toward Eisenmann's first or second sequence, which corresponds to the selectivity sequences of narrow pores with weak binding sites (Eisenman, 1962). Such pores rely heavily on ion dehydration to limit permeation to large ions (Figure 6). Small ions, which have a high charge density, need more energy to dehydrate. This gives them a larger effective radius, which decreases their relative permeability (Figure 6).

**Table 1:** **Selectivity sequences of various gating pores**.

**Channel**	**Mutation**	**Location**	**Selectivity**	**References**
*dShaker*	R362C	R1	Guanidium > Cs > K > Li	Tombola et al., 2005
rNa_v_1.4	R666G	DII/R2	Cs ~ K > Na ~ Li > TEA ~ NMDG	Sokolov et al., 2007
*N.at-*K_v_3.2	None^*^	None^*^	K > Cs ~ Guanidium > Na ~ Ba	Klassen et al., 2008
hH_v_1	R211S	R3	Guanidium > Li > H > Cs ~ K ~ Na	Berger and Isacoff, 2011
rNa_v_1.4	R1125Q	DIII/R2	K > Na >> NMDG	Francis et al., 2011

* *For the gating pore of N.at-K_v_3.2, no substitution was required to induce a gating pore*.

NMDG^+^ (N-Methyl-D-Glucamine) and TEA^+^ (Tetra-Ethyl Ammonium), two cations that are larger than K^+^, have been reported to flow through gating pores (Sokolov et al., 2007; Francis et al., 2011). Interestingly, the conductance values for NMDG^+^ and TEA^+^ are lower than for K^+^. This seems to contradict the hypothesis that gating pores typically follow Eisenmann's first two selectivity sequences. Once dehydrated, NMDG^+^ and TEA^+^ would either be small enough or too big to flow through this permeation pathway. In this case, gating pores would be less permeable to K^+^ than to NMDG^+^ or TEA^+^ since their charge density is lower (Figure 6). However, the studies reporting that NMDG^+^ and TEA^+^ flow through gating pores were both performed with extracellular solutions adjusted to pH 7.4. It is thus possible that the currents were the result of the permeation of H^+^ rather than NMDG^+^ or TEA^+^.

It has been reported that some mutations involving histidine substitutions in the *Shaker*, Na_v_1.4, and Na_v_1.5 channels yield gating pores that are selective to H^+^ alone (Starace and Bezanilla, 2004; Campos et al., 2007; Struyk and Cannon, 2007; Gosselin-Badaroudine et al., 2012a,b). It is noteworthy that the permeation of H^+^ through VSDs is postulated to occur via a Grotthus hopping mechanism via water wires on each side of the membrane (Starace and Bezanilla, 2004).

Indirect evidence for the flow of anions through mutant H_v_1 channels has recently been reported (Musset et al., 2011). It would thus be interesting to investigate this type of permeation in other VSDs.

#### Conductance of gating pores

Several data sets describe the conductance of gating pores (Table 2) (Cherny et al., 2003; Starace and Bezanilla, 2004; Sokolov et al., 2007, 2008, 2010; Struyk and Cannon, 2007; Tombola et al., 2007; Struyk et al., 2008; Francis et al., 2011; Gosselin-Badaroudine et al., 2012a,b). Given the diversity of the gating pores investigated and the methods used to carry out the investigations, no consensus seems to have emerged concerning the conductance of gating pores. To compare the conductance values obtained from different studies, we converted all the values into Siemens (Table 2). In these cases, some assumptions had to be made. At voltages of -140 mV and lower, the assumption was that the open probability for gating pores at hyperpolarizing voltages is 1. Given that, at this voltage, the current follows Ohm's law, the conductance should be maximal, and the driving force should be the only parameter impacting the value of the current value. Nevertheless, there is evidence that the open probability for gating pores might not be 1 in such cases (Tombola et al., 2007). Assuming unitary conductances of 25 pS and 22 pS for the alpha pores of the Na_v_1.4 and Na_v_1.5 channels, respectively (Gellens et al., 1992; Chahine et al., 1994a), we calculated the corresponding conductance values based on the gating pore currents measured, the voltages at which they were measured, and the solutions used (Table 2). The values measured and calculated span a wide range (0.17–1060 fS for gating pores created with single mutations and up to 3400 fS for gating pores created with multiple mutations) (Table 2). Interestingly, major discrepancies have been reported in the values of gating pore conductances of the Na_v_1.4/R666G mutant channel using similar techniques (Sokolov et al., 2007, 2008, 2010; Struyk and Cannon, 2007; Struyk et al., 2008). The discrepancies may be mostly due to the different mutations and the wide variety of proteins (Table 2).

**Table 2:** **Conductance values reported for various gating pores**.

**Channel**	**Mutation**	**Location**	**Conductance**	**Ion used for measurement**	**Evaluation mode**	**References**
*dShaker*	R362H	R1	40 fS	H^+^	Fluctuation analysis	Starace and Bezanilla, 2004
*dShaker*	R362C and R362C/E283D	R1 and S2/E1	100–600 fS	K^+^	Iω /Iα	Tombola et al., 2005
*dShaker*	A359G/R362S	R0 and R1	1410 fS	K^+^	Fluctuation analysis	Tombola et al., 2007
*dShaker*	R362S/E283D/S357C/M356D	R1, S2/E1 and others	3420 fS	K^+^	Fluctuation analysis	Tombola et al., 2007
rNa_v_1.4	R666G	DII/R2	530 fS	Na^+^	I_ω_ /I_α_	Sokolov et al., 2007
rNa_v_1.4	R663H	DII/R1	0,17 fS	H^+^	I_ω_ /Q_on_	Struyk and Cannon, 2007
rNa_v_1.4	R666H and R666G	DII/R2	34 fS	not clear (H^+^ and/or Na^+^)	I_ω_ /Q_on_	Struyk et al., 2008
rNa_v_1.4	R669Q, R669G and R669W	DII/R3	1060 fS	Na^+^	I_ω_ /I_α_	Sokolov et al., 2008
rNa_v_1.4	R666G	DII/R2	530 fS	Na^+^	I_ω_ /I_α_	Sokolov et al., 2010
rNa_v_1.4	R1125Q	DIII/R2	34 fS	Na^+^	I_ω_ /Q_on_	Francis et al., 2011
hNa_v_1.5	R219H	DI/R1	400 fS	H^+^	I_ω_ /I_α_	Gosselin-Badaroudine et al., 2012b
rNa_v_1.4	R219H, R663H and R1125H	DI/R1, DII/R1 and DIII/R2	210–420 fS	H^+^	Iω /Iα	Gosselin-Badaroudine et al., 2012a
hH_v_1	None^*^		38–400 fS	H^+^	Fluctuation analysis	Cherny et al., 2003

Evaluation modes:

*Fluctuation analysis: Analysis of the variance of the current as per Sigworth (1980)*.

*I_ω_/Q_on_: Normalization of the gating pore current to the observed gating charge*.

*I_ω_/I_α_: Normalization of the gating pore current to the maximum current of the alpha pore*.

* *For hH_v_1, the neutral residue in the R4 position appears to be involved in the creation of a conduction pathway through the VSD*.

The conductance of pores created by arginine-to-histidine mutations should be treated as special cases since their permeation mechanism is different. Arginine-to-histidine mutations yield gating pore currents that are highly selective for H^+^. The conductance of these pores would be primarily modulated by the accessibility of the histidine residue to the GCTC. When the histidine residue is very close to the GCTC and the water crevices are deep, the pores would display high conductances.

## Naturally occurring gating pores

### Gating pore currents in physiological conditions

When observed in living organisms, gating pore currents are mainly described as a pathogenic process associated with naturally occurring mutations. Nevertheless, they can be found in few physiological conditions. The H_v_1 channel (also called HVCN1 or VSOP) is structurally composed of four TM segments with cytoplasmic C- and N-termini (Capasso et al., 2011) (Figure 1). Interestingly, this protein does not feature the S5–S6 motif frequently associated with selective ion conduction (Figure 1). However, this channel is similar to the VSDs of other VGICs (Figures 1, 2) (Ramsey et al., 2006; Sasaki et al., 2006). It has been reported that H^+^ ions can permeate the protein via the VSD using a proton-wire mechanism (Nagle and Morowitz, 1978; Decoursey, 2013). According to the definition of gating pore currents put forward by Tombola et al. (2005), H_v_1 currents can be considered as gating pore currents. Physiologically, the activation of this channel causes an efflux of cytoplasmic H^+^ (Decoursey, 2003).

H_v_1 plays key roles in numerous processes such as phagocytosis, spermatozoa maturation, and B cell activation (Capasso et al., 2011). The activation of H_v_1 results in the regulation of NADPH (Nicotinamide Adenine Dinucleotide Phosphate) function, ensuring an optimal pH in phagocytosis vesicles (Decoursey, 2003). The H_v_1 channel is also involved in cardiac function (El Chemaly et al., 2006). The involvement of gating pore currents mediated by H_v_1 in a wide array of functions highlights its physiological importance.

Naturally occurring gating pore currents in platyhelminthes (flat worms) are another example of the involvement of gating pores in physiological functions. A K^+^ channel expressed in flat worms (*N*.*at* − *K*_v_3.2) naturally conducts ions through the VSD (Klassen et al., 2008). These channels display unusual properties compared to other K_v_ channels of the *Shaw* family (K_v_3 family) (Klassen et al., 2006). Surprisingly, WT N.at-K_v_3.2 channels exhibit inward-rectifier currents that are activated by hyperpolarization. The *N.at-K_v_3.2* protein structure is composed of six TM. Its pore region is very similar to that of other K_v_ channels. However, the S4 helix features two atypical residues (a histidine and a glycine) in positions where the first and third arginines are usually found (Figure 2) (Klassen et al., 2008). This enables the permeation of cations through the VSDs of *N.at-K_v_3.2* channels due to the lack of positively charged residues on S4 (Figure 2). Hyperpolarization-activated currents in these channels are gating pore currents. In this flat worm, gating pore currents would be involved in their physiological functions. Unfortunately, their exact physiological role is unknown.

### Are gating pores a feature of evolution?

Klassen et al. proposed that gating pores in *N.at-K_v_3.2* have evolved to produce a non-selective cation channel resulting in the creation of a new molecular property (Klassen et al., 2008) adapted for a specific physiological need. From an evolutionary point of view, VGICs could be the result of the evolution of a unique TM segment (an S3-like segment) (Kumanovics et al., 2002) that is responsible for sensing membrane curvature. This unique TM could then have evolved into a VSD, which would have become fine-tuned for voltage sensing before coupling with pre-existing pores. This assembly would form the VGIC (Kumanovics et al., 2002). Such an assembly is in agreement with the discovery of other channels such as H_v_1, which are only composed of a VSD (Ramsey et al., 2006; Capasso et al., 2011) and voltage-dependent phosphatases such as *Ciona intestinalis* voltage-sensitive phosphatase (Ci-VSP) (Murata et al., 2005). These phosphatases are examples of a different evolutionary pathway of the VSD motif. Indeed, depending on the subtype of protein assembly, different physiological functions can appear. In this case, Ci-VSP is directly coupled to and activated by a VSD (Murata et al., 2005). Later in the evolutionary process, four subunits composed of a VSD and a pore domain fused to form Na_v_ and Ca_v_ channels (Kumanovics et al., 2002). Even if the naturally occurring gating pores in *N.at-K_v_3.2* can be considered as evolutionary precursors of VSDs, they are (together with H_v_1) the only known naturally occurring gating pores. Most known gating pores are *in vitro* creations, or are of pathological origin, which would likely exert negative selection pressure and decrease the probability of conserving these gating pores throughout the evolutionary process.

## Pathologies linked to gating pore currents

### Periodic paralysis

#### Hypokalemic periodic paralysis

**Figure 7:**
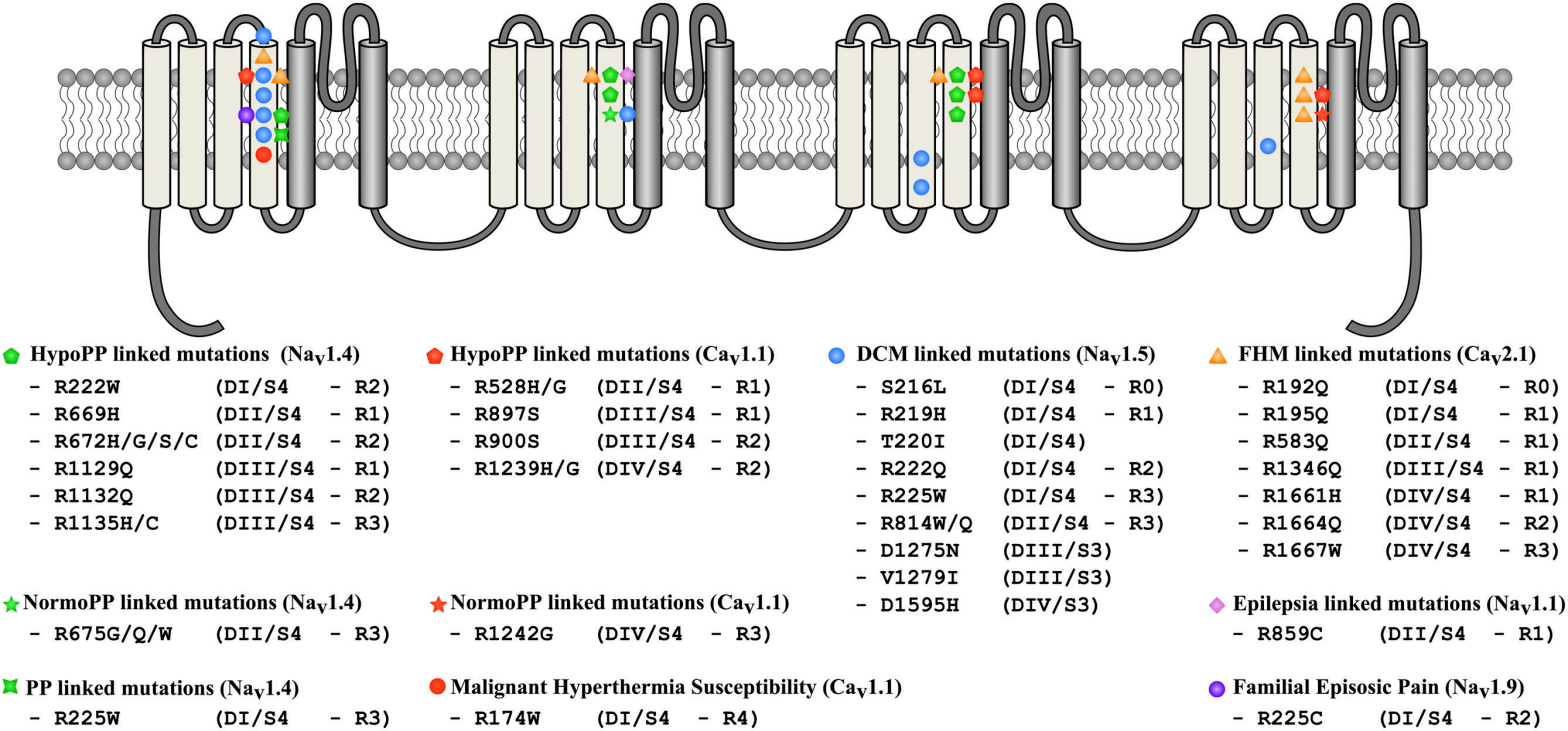
**Locations of mutations potentially associated with the creation of gating pores in Na_v_ and Ca_v_ channels**. The 24 TM segments are shown on a 2-D representative structure of a Na_v_ or a Ca_v_ channel. Mutations associated with DCM are indicated by blue circles, periodic paralysis phenotypes linked to Na_v_1.4 mutations are indicated by green symbols. Periodic paralysis phenotypes linked to Ca_v_1.1 mutations are indicated by red pentagons and stars. The epilepsia-linked mutation is indicated by a purple diamond and familial hemiplegic migraine mutations, by orange triangles. Malignant hyperthermia susceptibility and familial episodic pain linked mutations are respectively indicated by red and purple circle.

Hypokalemic periodic paralysis (HypoPP) is a rare autosomal dominant disorder characterized by recurrent attacks of paralysis in the presence of low serum K^+^ (< 3 mEq/L). Paralysis episodes can be triggered by carbohydrate-rich meals, exercise, exposure to cold, fever, or emotional stress (Cannon, 2006; Sung et al., 2012). The current explanation for these paralysis attacks is that the resting membrane potential (V_REST_) changes from −85 mV to −60 mV under hypokalemic conditions, leading to muscle fiber inexcitability (Rudel et al., 1984; Jurkat-Rott et al., 2009). To date, 16 mutations on the *CACNA1S* and *SCN4A* genes have been shown to cause HypoPP-1 and HypoPP-2, respectively (Figure 7). These two genes encode Ca_v_1.1 and Na_v_1.4, respectively. Missense mutations have been found on their S4 segments (six for *CACNA1S* and ten for *SCN4A*, Figures 2, 6) (Jurkat-Rott et al., 2012; Groome et al., 2014). Mutations on *CACNA1S* account for approximately 60% of HypoPP cases while mutations on *SCN4A* account for 10% (Tricarico and Camerino, 2011). Initial studies have revealed that missense mutations cause some discrete changes in currents carried by the mutated channels (Lapie et al., 1996; Morrill et al., 1998; Jurkat-Rott et al., 2000). Interestingly, mutations causing HypoPP are reported to result in a number of changes to the biophysical properties of the channels. For example, some HypoPP mutations on Ca_v_1.1 may solely affect current density while others may result in slower channel activation (Lapie et al., 1996; Morrill et al., 1998). On the other hand, HypoPP mutations on Na_v_1.4 are reported to lead to divergent biophysical defects since they result in enhanced or reduced channel inactivation (Jurkat-Rott et al., 2000). Nevertheless, these changes cannot fully explain the depolarization observed during paralysis episodes (Cannon, 2010). The specific location of the mutations on the S4 segments (Figure 7) of the two genes led the researchers to focus on finding a common pathologic mechanism. Gating pore currents were described as the common mechanism leading to the muscular pathology (Sokolov et al., 2007; Struyk and Cannon, 2007). At this juncture, two types of mutations have been described: mutations leading to the creation of a cationic leak (Sokolov et al., 2007) and mutations leading to the creation of an H^+^-specific leak (arginine-to-histidine substitutions) (Struyk and Cannon, 2007).

**Figure 8:**
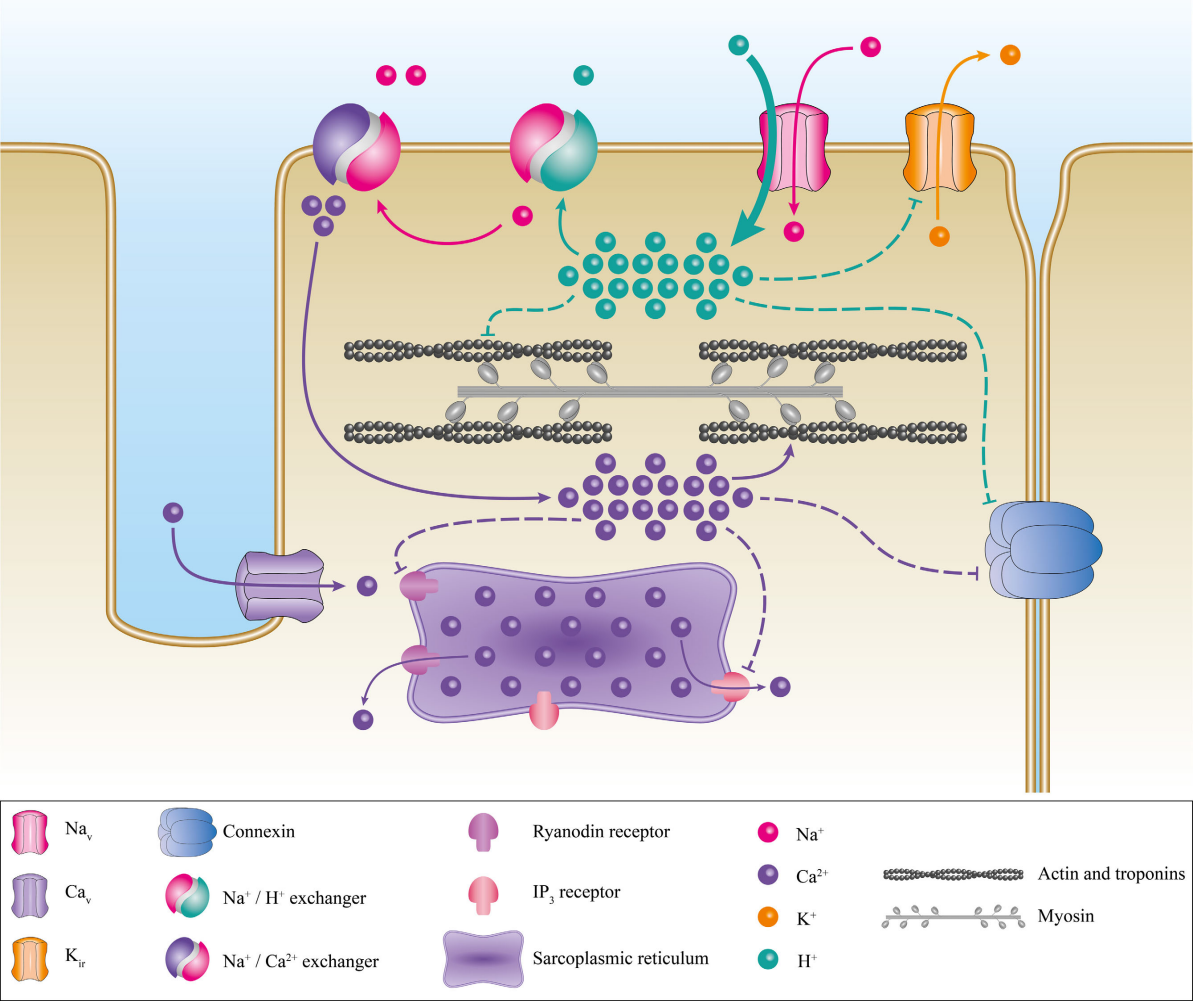
**Proposed pathogenic mechanism associated with the creation of a gating pore**. Schematic representation of a myocyte with its main ionic channels and exchangers. The contractile proteins, the sarcoplasmic reticulum, and connexins are in gray, purple, and blue, respectively. The appearance of a gating pore current induces a disequilibrium of ionic homeostasis through the activation of several exchangers such as Na^+^/H^+^ and Na^+^/Ca^2+^. This would result in a Ca^2+^ overload. The disequilibrium then destabilizes the resting membrane potential, connexin activity, and excitation-contraction coupling. The pathogenic mechanism resulting in the creation of a cation-selective gating pore current is not shown here. However, it would be similar to the pathogenic mechanism resulting in a proton-selective gating pore current. A cation-selective gating pore current would inhibit K_ir_ channels, thus increasing the resting membrane potential. A gating pore current would inhibit the Na^+^/H^+^ exchanger and activate the Na^+^/Ca^2+^ exchanger. This would result in acidosis and a Ca^2+^ overload.

Several processes have been proposed to explain this pathology. Primarily, the elevation of V_REST_ induces the inactivation of Na_v_1.4 thus abrogating the ability to excite muscle fibers. The V_REST_ of a muscular fiber is mainly set by K_ir_ (Inward Rectifier Potassium Channels) (Hibino et al., 2010; Wu et al., 2011). In severe hypokalemia (<1 mM), K_ir_ activity is reduced. In HypoPP, the inward Na^+^ or H^+^ leak induced by mutations of Na_v_1.4 or Ca_v_1.1 inhibits K_ir_ currents under relatively low kalemia conditions (2.5–3 mM K^+^) (Figure 8). The reduction in the outward K^+^ current then causes an elevation in V_REST_ (Struyk et al., 2008; Jurkat-Rott et al., 2009; Cannon, 2010; Tricarico and Camerino, 2011; Wu et al., 2011). Gating pore currents also induce a Na^+^ overload in muscle fibers, which has already been reported by Jurkat-Rott et al. (Jurkat-Rott et al., 2009). The Na^+^ overload further destabilizes ionic homeostasis (Figure 8). Several membrane exchangers can also be activated, including Na^+^/K^+^ ATPase, Na^+^/H^+^, Na^+^/Ca^2+^, Na^+^/Lac^−^, and Na^+^/bicarbonate. Na^+^/H^+^ exchangers usually extrude H^+^ from the cell to regulate the internal pH. However, this exchanger may work in reverse mode (Hilgemann et al., 2006), which would result in intracellular acidosis. In addition, the exact downstream events resulting from a Na^+^ overload are not fully understood. Nevertheless, detrimental consequences have been described (Pieske et al., 2003; Maack et al., 2006; Kohlhaas et al., 2010). A Na^+^ overload may also increase the mitochondrial production of reactive oxygen species (Kohlhaas et al., 2010), which would result in an increase in oxidative stress, leading to cellular remodeling as well as excitation-contraction uncoupling, and electrical disturbances (Pieske et al., 2003; Maack et al., 2006; Kohlhaas et al., 2010).

Mutations that induce H^+^-selective gating pores are linked to the same phenotype as mutations that induce cation-selective gating pores (Struyk et al., 2008) (Figure 8), indicating that they have similar downstream consequences although through slightly different mechanisms. This could be explained by the activity of the exchangers described previously. H^+^ leaks and intracellular acidification can have specific impacts on muscular fibers, including the blockade of K_ir_ (Figure 8) (Tricarico and Camerino, 2011), which would increase the depolarization process. Acidification can also lead to connexin uncoupling (Bukauskas et al., 2001) and the impairment of excitation-contraction coupling by decreasing the affinity of troponin C for Ca^2+^ (Figure 8) (Fabiato and Fabiato, 1978; Ball et al., 1994; Palmer and Kentish, 1994; Parsons et al., 1997). HypoPP muscle fibers display certain peculiarities such as the formation of vacuoles (vacuolar myopathy) and tubular aggregates (Sternberg et al., 2001; Jurkat-Rott et al., 2009; Tricarico and Camerino, 2011; Wu et al., 2011). Wu et al. also reported that sarcoplasmic triads are dilated in their HypoPP knock-in mouse model (Wu et al., 2011, 2012). All these morphological changes, while they have not been studied in depth, attest to the diverse impacts of gating pore currents (H^+^-specific or not). Unfortunately, the processes leading to morphological changes are unknown.

Wu et al. recently described the link between HypoPP-1 and mutations on Ca_v_1.1. They used a knock-in mouse model and reported that gating pore currents are induced in Ca_v_1.1 channels featuring an arginine-to-histidine mutation in the S4 segment (Wu et al., 2012). Their study confirmed that the pathogenic process leading to HypoPP-1 may be similar to the process described for HypoPP-2.

#### K^+^-sensitive normokalemic periodic paralysis

At physiological K^+^ concentrations, some patients express a variant of HypoPP called K^+^-sensitive normokalemic periodic paralysis (NormoPP). Vicart et al. identified three new mutations of the third arginine of the S4 segment of DII of the human Na_v_1.4 protein (R675G/Q/W) (Figure 7) (Vicart et al., 2004). A biophysical study of these mutants showed that gating pore currents appeared under depolarized conditions while the usual biophysical properties were only slightly impacted (Sokolov et al., 2008). The main characteristic of these gating pore currents is an ionic leak when Na_v_1.4 is in the activated state (mostly K^+^ efflux) and slow-inactivated state (mostly Na^+^ influx) (Sokolov et al., 2008). The Na^+^ influx during the slow-inactivated state occurs when the membrane potential is hyperpolarized. The mutations may stabilize the S4 segment in the activated state (Sokolov et al., 2008). Vicart et al. attributed the pathological mechanism to the large increase in the Na^+^ influx in the slow-inactivated state. Recently, the R1242G mutation on Ca_v_1.1 channels has also been associated with the development of NormoPP (Fan et al., 2013). Gating pore currents have been recorded in the depolarized state, suggesting a common pathological mechanism. The study also confirmed that an inward gating pore current can be recorded under hyperpolarized voltages after a long depolarization period (Fan et al., 2013). Interestingly, the R1135H/C mutations have recently been associated to the development of HypoPP phenotype (Groome et al., 2014). These mutations also induce gating pore activated by depolarization. Furthermore, the study reveals the presence of gating pore current under hyperpolarized conditions probably associated with the freezing of the VSD after long depolarizations.

Intriguingly, Lee et al. described a patient affected by myotonia that was triggered by cold exposure and that was associated with transient weakness (Lee et al., 2009). The genetic study revealed the presence of a *SCN4A* mutation that changed the third R of DI of the S4 segment to a W (R225W) (Figures 2, 6). While the clinical phenotype was not classified as HypoPP or NormoPP, the mutation was strongly associated with the development of muscular weakness. The creation of a gating pore, which is activated by depolarization due to the mutation, is highly probable (Figure 5C) and may be the cause of the pathology.

### Mixed arrhythmias associated with dilated cardiomyopathy

DCM is the most common cardiac disorder. It is characterized by left ventricular dilatation (>117%) and systolic dysfunction (ejection fraction inferior to 45%) (Richardson et al., 1996; Taylor et al., 2006; McNair et al., 2011). DCM has a prevalence of 1 in 2500 and an annual incidence of 7 in 100,000 a year (Taylor et al., 2006). Some 20–48% of DCM cases are classified as familial DCM (Hershberger and Siegfried, 2011). To date, 33 genes, most of which code for cytoskeleton or contractile proteins, have been associated with familial DCM (Hershberger and Siegfried, 2011). McNair et al. recently reported that *SCN5A* is linked to 1.7% of familial DCM cases (McNair et al., 2011). The *SCN5A* gene codes for the cardiac Na_v_1.5 channel, which is responsible for the initiation of the action potential in the heart. Its role is comparable to that of Na_v_1.4 in skeletal muscles. So far, 18 *SCN5A* mutations have been associated with the development of severe arrhythmias and heart dilatation. Ten of the mutations are located on the VSD (S216L, R219H, T220I, R222Q, R225W, R814W, R814Q, D1275N, V1279I, and D1595H) (Figure 7) (Bezzina et al., 2003; McNair et al., 2004; Frigo et al., 2007; Ge et al., 2008; Hershberger et al., 2008; Hedley et al., 2009; McNair et al., 2011; Gosselin-Badaroudine et al., 2012b; Laurent et al., 2012). While Na_v_1.5 dysfunctions are commonly linked to rhythm disturbances such as type 3 Long QT Syndrome (LQTS), Brugada Syndrome (BrS), Sick Sinus Syndrome (SSS), or conduction defects (Amin et al., 2010), dysfunctions of this protein have also been linked to a morphological defect (Gosselin-Badaroudine et al., 2012b). Interestingly, the phenotype observed in cases of DCM-linked *SCN5A* mutations is a combination of complex arrhythmias and cardiac dilatation. In addition, while mutated Na_v_1.5 proteins cause different biophysical defects, the clinical phenotypes reported clearly show major similarities (Table 3). We recently linked the R219H mutation on the *SCN5A* to mixed arrhythmias and the DCM phenotype, although the classic biophysical properties of the mutant protein are not affected (Table 3) (Gosselin-Badaroudine et al., 2012b). We described a 29-year-old patient who suffered from cardiac dilatation associated with complex arrhythmias including bradycardia, atrial flutter, atrio-ventricular block, and tachycardia (Table 3) (Gosselin-Badaroudine et al., 2012b). Since the mutated residue was the first highly conserved arginine on the S4 segment of DI (Figures 2, 6), the lack of alterations to the biophysical properties of the channel led us to search for a gating pore current. Our study revealed that the R219H mutation induces a proton-specific gating pore current (Gosselin-Badaroudine et al., 2012b). Given the location of the other mutations associated with similar phenotypes (Figure 7), we propose that the generation of gating pore currents may be the common underlying mechanism that links all the phenotypes (Table 3) (Gosselin-Badaroudine et al., 2013). While all the mutations are not located on the S4 segment, mutations on the S1, S2, or S3 segment may allow the water crevices to communicate and induce gating pore currents (Figure 7).

**Table 3:** **Biophysical and clinical comparison of mutations associated with the development of arrhythmias and DCM**.

**Mutation**	**Biophysical defect**					**Clinical Phenotype**				**References**
	**Current density**	**Activation**	**Inactivation**	**Recovery**	**Kinetics**	**Atrial**	**Conduction system**	**Ventricular**	**Other**	
S216L DI/R0	≈	≈	+4.7 mV	≈	I Fast	AFib			SSS, LQT	Wang et al., 2007; Hershberger et al., 2008; Hedley et al., 2009; Olesen et al., 2012
R219H DI/R1	≈	≈	≈	≈	≈	AFL	AVB	Tach, PVC	Brad	Gosselin-Badaroudine et al., 2012b
T220I DI/S4	↓	≈	−4.4 to −5.6 mV	Slow	I slow	AFib, AI	AVB, BBB	Tach	Sync, SSS	Benson et al., 2003; Olson et al., 2005; Gui et al., 2010a
R222Q DI/R2	↓,≈	−6.3 to −13 mV	−4 to −7.3 mV	Slow	A Fast, I Fast	AFib, PAC, AFL	AVB, BBB	Tach, PVC	Brad	Cheng et al., 2010; Laurent et al., 2012; Mann et al., 2012; Nair et al., 2012
R225W DI/R3	↓	+14 mV	+11 mV	≈	≈		AVB	Tach		Bezzina et al., 2003
R814Q DII/R3	ND	ND	ND	ND	ND		BBB	Tach, PVC		Frigo et al., 2007
D1275N DIII/S3	≈,↓	≈, +3.1 mV	≈, −8 mV, +7.6 mV	≈, Fast	I Slow	AFib, AS, AFL, Brad, Tach	AVB, BBB	Tach, PVC		Groenewegen et al., 2003; McNair et al., 2004; Olson et al., 2005; Laitinen-Forsblom et al., 2006; Gui et al., 2010a,b; Watanabe et al., 2011
V1279I DIII/S3	ND	ND	ND	ND	ND	AFib	BBB	Tach		McNair et al., 2011
D1595H DIV/S3	≈	≈	−6.8 mV	Slow	I Slow	AFib			Brad	Olson et al., 2005; Nguyen et al., 2008

*↑, Increase; ↓, Decrease; ≈, No impact; A Fast, faster activation kinetics; AFib, atrial fibrillation; AFL, atrial flutter; AI, atrial inexcitability; AS, atrial standstill; A Slow, slower activation kinetics; AVB, first, second, or third degree atrio-ventricular block; BBB, incomplete right or left bundle branch block; Brad, bradycardia; I Fast, faster inactivation kinetics; I Slow, slower inactivation kinetics; LQT, long QT syndrome; ND, not determined; PAC, premature atrial contractions; PVC, premature ventricular contractions; SSS, sick sinus syndrome; Sync, syncope; Tach, tachycardia*.

In a recent review, Jurkat-Rott et al. classified the R225Q/W and R814Q *SCN5A* mutations as type 3 LQTS (Jurkat-Rott et al., 2012). They proposed that the substitution of these highly conserved arginines on the S4 segment (Figure 2) creates a gating pore. Interestingly, the original articles describing these mutations report that LQTS is associated with other arrhythmias and heart morphology abnormalities (Bezzina et al., 2003; Frigo et al., 2007). Here again, the presence of gating pores due to the mutations was not investigated.

The molecular mechanisms for the pathogenic process leading to the development of this complex pathology may be similar to the process observed in HypoPP (Figure 8). The H^+^ leak (or possibly non-specific cation leak) might imbalance the V_REST_ of cardiomyocytes and trigger the arrhythmias. Intracellular acidification may also have an impact on connexin conductance, uncoupling intercellular communication (Bukauskas et al., 2001). The cardiac remodeling may be due to the major imbalance in ionic homeostasis. Gating pore currents, combined with the activity of numerous exchangers, may thus cause significant changes in Na^+^, Ca^2+^, and H^+^ concentrations, which would decrease the affinity of troponin for Ca^2+^ (Figure 8). Cardiac contraction disturbances have been reported to result from alterations to the affinity of troponin for Ca^2+^ (Liu et al., 2012). This would lead to a reduction in contractile strength, which in turn would result in an impaired ejection fraction. Moreover, the uncoupling of sarcomeric proteins may have an impact on the cellular structure, leading to cardiac remodeling and DCM.

However, this cardiac remodeling may also be the result of weakened contractions in a background of normal blood pressure on the heart walls. Unfortunately, the morphological structure of cardiomyocytes has not been studied in great depth, which limits our understanding of the pathological processes. Lastly, gating pore currents caused by other Na_v_1.5 mutations (Figure 7) need to be recorded to confirm that gating pore currents are a common mechanism linked to the development of familial DCM.

### Peripheral nerve hyperexcitability

Peripheral nerve hyperexcitability (PNH), also known as neuromyotonia, is a motor neuron dysfunction with heterogeneous clinical symptoms. PNH has a prevalence of <1 in 1 million. Two main subtypes of PNH can be distinguished, i.e., the autoimmune and the non-autoimmune form. Among the non-autoimmune forms, 2 of the 3 subtypes of hereditary PNH are channelopathies. The most common symptoms include skeletal muscle overactivity, muscle twitching and painful cramps (Jurkat-Rott et al., 2010).

The KCNQ2 gene encodes the K_v_7.2 K^+^ channel which contributes to the neuronal non-inactivating M-current (Cannon, 2006). These K_v_7.2 channels are broadly expressed in the brain and the spinal cord (Schroeder et al., 1998; Dedek et al., 2001). In physiological conditions, K_v_7.2 channels are co-expressed with K_v_7.3 channels to form heterotetramers responsible for the M-current. The slow opening of these channels mainly sets the resting membrane potential and thus modulates the neuronal firing frequency (Hernandez et al., 2008). Most known mutations of the K_v_7.2 channel are associated with the development of neonatal epilepsy known as benign familial neonatal convulsion (BFNC). These K_v_7.2 mutations are mainly localized in the PD, the intra and cellular loops. The contribution of this channel to the disease manifestation could thus be due to its role in setting the neuronal resting membrane potential (Lerche et al., 2005).

Recent reports suggest that K_v_7.2 mutations in the channel's VSD may be associated with other clinical phenotypes (Dedek et al., 2001; Wuttke et al., 2007). The R207W and R207Q mutations result in the neutralization of the fourth conserved arginine of the VSD's S4 segment (R4W and R4Q). The R207W mutation has been linked to the development of a BFNC phenotype associated with PNH (Dedek et al., 2001). The similar R207Q mutation has been linked to PNH without BFNC (Wuttke et al., 2007).

The R207W biophysical characterization revealed that the mutation causes a depolarizing shift in the voltage-dependence and a drastic slowing of activation resulting in a loss of channel function. This would probably explain the BFNC and PHN phenotype (Dedek et al., 2001). When K_v_7.2 and K_v_7.3 were co-expressed to potentially mimic a probable physiological condition, this loss of channel function due to the R207W mutation is still observed (Dedek et al., 2001).

The investigation of the R207Q mutation also revealed a loss of channel function *via* similar mechanisms (Wuttke et al., 2007). However, this loss of channel function disappeared in conditions potentially mimicking the situation of a patient carrying the R207Q mutation (co-expression of K_v_7.2/R207Q, K_v_7.2/WT, and K_v_7.3 subunit) (Wuttke et al., 2007).

The localization of R207Q and R207W mutations led the researchers to suspect and then confirm the presence of gating pore current. Due to low K_v_7.2 channel expression, the presence of gating pore current was investigated in homologous K_v_7.4 channels. It was thus found that reproducing the R207Q or R207W mutations caused the creation of a gating pore. Since this channel is similar to the K_v_7.2 channel, it is reasonable to assume that the gating pore current observed in K_v_7.4 would be similar in K_v_7.2 channels.

Therefore, the common PNH phenotype observed could be due to the gating pore current induced by the R207W/Q mutations. Indeed, in conditions close to the physiology, the R207Q mutation did not seem to change channel's function while the R207W mutation induced a loss of channel function (Wuttke et al., 2007). This might thus explain why the BFNC phenotype is observed with the R207W mutation whereas the R207Q mutation only causes PNH.

The pathophysiological process could be similar to the one observed with the R675W mutation (R3W) of the Na_v_1.4 isoform causing NormoPP previously discussed. The Na^+^ leak caused by gating pore currents could facilitate the generation of action potentials by lowering the threshold, thus leading to neuronal hyperexcitability.

### Other gating pore linked pathologies

To date, HypoPP, NormoPP, arrhythmic familial DCM and PNH are the only pathologies that have been clearly associated with gating pore currents. However, recent reports, based solely on the location of the mutation, have indicated that other pathologies may be linked to gating pore currents. A total of 28 mutations on the S4 segments of Na_v_, Ca_v_, and K_v_ have been described. These mutations cause six other pathologies (LQTS, familial hemiplegic migraine, epilepsy, benign familial neonatal infantile seizures, familial episodic pain, and malignant hyperthermia susceptibility) (Figure 7) (Scalmani et al., 2006; Striessnig et al., 2010; Jurkat-Rott et al., 2012; Zhang et al., 2013). However, the association between gating pores and these pathologies are hypothetical and more studies are required to confirm the presence of gating pores. As such, these assumptions should be treated with caution. Similar assumptions have been made about the R1448C and R1448H Na_v_1.4 mutations (R1–S4/DIV) (Sokolov et al., 2007). These mutations have been linked to the occurrence of paramyotonia congenital (Chahine et al., 1994b). Gating pore currents should appear, based on the location of the mutation (first arginine on S4 of DIV). However, the R1448C mutation does not induce a gating pore current (Francis et al., 2011), which was recently confirmed by Gosselin-Badaroudine et al. (2012a). It is thus far from certain that a given mutation will induce a gating pore current based solely on the site of the mutation.

## Pharmacology

### Blockers as research tools

A few gating pore blockers have been tentatively identified. However, due to the wide diversity of the immediate environment of gating pores, no universal blocker has yet been identified.

Divalent cations such as barium (Ba^2+^), zinc (Zn^2+^), and Ca^2+^ are effective in the millimolar (mM) range (Table 4). These cations block gating pores created by the arginine-to-glycine substitution of the second arginine of the S4 segment in DII of Na_v_1.4 (R666G) (Sokolov et al., 2007). Nickel (Ni^2+^), cadmium (Cd^2+^), and barium (Ba^2+^), on the other hand, do not block gating pores created by the R663H mutation in Na_v_1.4 (Table 4) (Struyk and Cannon, 2007). Possible explanations include the location of the mutation and the nature of the gating pore current since gating pores induced by the R666G mutation conduct monovalent cations while the R663H mutant channel specifically conducts H^+^.

**Table 4:** **Ions and molecules tested as gating pore blockers**.

**Channel**	**Mutation**	**Location**	**Ions**		**Guanidinium derivatives**		**References**
			**Block**	**No effect**	**Block**	**No effect**	
*dShaker*	R362C/E283D	R1/E1	Mg^2+^		Ethyl-guanidium		Tombola et al., 2005
rNa_v_1.4	R666G	DII/R2	Ca^2+^, Zn^2+^, Ba^2+^				Sokolov et al., 2007
rNa_v_1.4	R663H	DII/R1		Li^+^, Ni^2+^, Cd^2+^, Ba^2+^			Struyk and Cannon, 2007
rNa_v_1.4	R666G	DII/R2		Ca^2+^, Zn^2+^, Ba^2+^			Struyk et al., 2008
*N.at-*K_v_3.2	None^*^	None^*^		Ba^2+^			Klassen et al., 2008
rNa_v_1.4	R666G	DII/R2	Ca^2+^, Zn^2+^, Y^3+^, Ba^2+^, La^3+^, Yb^3+^, Lu^3+^, Hf^4+^, Tl^3+^		1-(2,4-xylyl) guanidine-carbonate	Ethyl- guanidium	Sokolov et al., 2010
hH_v_1	R211S	R3	Zn^2+^				Berger and Isacoff, 2011
rNa_v_1.4	R1125Q	DIII/R2	Ni^2+^, Zn^2+^	Ca^2+^, Ba^2+^			Francis et al., 2011
hNa_v_1.5	R219H	DI/R1		Ni^2+^, Zn^2+^, Cd^2+^,La^3+^		Ethyl- guanidium	Gosselin-Badaroudine et al., 2012b

* *For N.at-K_v_3.2, no substitution was required to induce a gating pore*.

Conflicting results have been published. Sokolov et al. studied the R666G mutation in Na_v_1.4 and reported that gating pores can be blocked by divalent cations (Sokolov et al., 2007) while Struyk et al. reported that divalent cations do not block gating pores (Table 4) (Struyk et al., 2008). This result was the subject of some debate until it was discovered that divalent cations do indeed block gating pores created by the R666G mutation in a voltage-dependent manner (Sokolov et al., 2010). Trivalent and quadrivalent ions (gadolinium Gd^3+^, ytterbium Yb^3+^, lanthanum La^3+^, lutetium Lu^3+^, yttrium Y^3+^, titanium Ti^3+^, and hafnium Hf^4+^) have also been reported to block gating pores, but in a voltage-independent manner (Sokolov et al., 2010).

The arginine residue can be considered as a natural gating pore blocker since gating pore currents appear following the substitution of the arginine on the S4 segment of VSDs (Figures 3, 4). Some researchers are thus investigating molecules derived from the side chain of arginine as blockers. The arginine side chain contains a guanidine. Interestingly, guanidinium ions permeate through gating pores while guanidinium derivatives such as ethylguanidinium and 1-(2,4-xylyl) guanidine carbonate have been reported to block gating pores (Table 4) (Tombola et al., 2005; Sokolov et al., 2010).

Gating pore blockers can be useful research tools, but the variability in published data is puzzling. Several investigators have reported that divalent and trivalent cations can block gating pores (Sokolov et al., 2007, 2010; Wu et al., 2011), while others have reported the opposite (Table 4) (Struyk and Cannon, 2007; Francis et al., 2011; Gosselin-Badaroudine et al., 2012b). This discrepancy may be due to differences in the environments surrounding the gating pores. However, investigators studying the same mutation have reported opposite results (Sokolov et al., 2007; Struyk et al., 2008) In this case, the discrepancy may be due to differences in the experimental methods (Sokolov et al., 2010). The voltage-dependence of the block may also explain the discrepancy. New studies are needed to clarify this issue.

Gating modifier toxins can also be useful in fundamental research. These toxins can stabilize the VSDs of various ion channels in their resting or activated state. Six toxin binding sites (sites 1–6) have been identified in Na_v_, with site 4 located in the VSD of DII (Cestele and Catterall, 2000). Toxins that affect site 4 can be used to modulate ion permeation through gating pores. For example, toxins in the same category as β-scorpion and β-spider toxins, including Css4 (*Centruroides suffusus suffusus* toxin 4), Tsγ (*Tityus serrulatus gamma* toxin), Lqhβ 1 (*Leiurus quinquestriatus hebraeus Beta* 1 toxin), AahIT (*Androctonus australis* Hector Insect Toxin), and Magi 5 (*Macrothele gigas* toxin 5) stabilize VSDs in their activated state (Martin et al., 1987; Chahine et al., 1998; Possani et al., 1999; Corzo et al., 2003; Stevens et al., 2011). In contrast, toxins such as μ-Oconotoxins (MrVIA and MrVIB) as well as HWTX-IV (Huwentoxin-IV) have the same binding site as β-scorpion toxins (site 4), but stabilize VSDs in their resting state (Leipold et al., 2007; Xiao et al., 2008).

### Targeting VSDs as a therapeutic approach

None of the reported gating pore blockers has been shown to be clinically relevant. When the pathology is known to be caused by gating pore currents, patients only benefit from symptomatic treatment targeting the downstream consequences of the mutation (paralysis or arrhythmias). Before a gating pore blocker can be used on a given patient, its effectiveness has to be verified *in vitro* on the patient's specific mutation. This is of critical importance since there are no known universal gating pore blockers. Most drugs target the central alpha pore. However, the fact that gating pore currents play a role in pathogenic processes will help in the development of new therapies that target VSDs. For example, molecules such as NH29 (a diclofenac derivative), a gating modifier that promotes the opening of the K_v_7.2 channel (Peretz et al., 2007, 2010), could be a starting point for the development of new drugs.

Given the diversity of the mutations, targeting VSDs could be an effective strategy for sidestepping the variability of the mutations (nature of the mutations, nature of the pathological current, voltage dependence). Modulating VSDs would stabilize a given mutated S4 segment in the activated or resting state in order to prevent water crevices from communicating and, as such, prevent gating pore currents. For example, β-scorpion toxins bind to site 4 on the Na_v_ channel and stabilize the S4 segment of DII in its activated state (Catterall et al., 2007). Unfortunately, the precise impact of each S4 segment and their possible cooperation in the gating of the alpha pores of the Na_v_ and Ca_v_ channels need to be clarified. Maintaining the gating properties and alpha pore functions of channels is a major challenge for developing therapies to specifically treat gating pore current-induced pathologies. So far, only 1-(2,4-xylyl) guanidine carbonate (Table 4) appears to be an efficient, specific blocker that could be used on most gating pores, especially since it does not modify the gating properties of the alpha pore (Sokolov et al., 2010). It would be very interesting to test 1-(2,4-xylyl) guanidine carbonate in the two transgenic animal models of HypoPP (Wu et al., 2011, 2012) in which the phenotype is caused by a Na_v_1.4 mutation in one model and a Ca_v_1.1 mutation in the other. It would also allow the blocker to be tested on different gating pores.

Developing approaches to modulate VSDs to treat channelopathies should also be considered. Even if such studies reveal that changing gating properties should be avoided in the case of gating pore currents, they could lead to new therapeutic avenues for treating cell excitability disorders and channelopathies such as LQTS and BrS. In addition, therapies that prevent the activation of specific Na_v_ (Na_v_1.7 or Na_v_1.8) channels might also be valuable in treating pain while avoiding the addictive side effect of currently available drugs.

## Perspectives and conclusions

The recent discovery of gating pore currents provides a fresh perspective in the field of electrophysiology. Gating pores are a promising new tool for the investigation of VSD structures and functions. Only a few crystal structures of VGICs have been reported. Moreover, the structures of only six TM channels have been elucidated (Jiang et al., 2003; Long et al., 2005, 2007; McCusker et al., 2012; Payandeh et al., 2012; Zhang et al., 2012). Gating pore currents are thus a powerful tool for investigating similarities and differences between VSDs. Simulations based on existing crystal structures have been used to understand and predict the complex tridimensional structures of Na_v_ and Ca_v_ channels (Tao et al., 2010; Delemotte et al., 2011; Khalili-Araghi et al., 2012). Gating pore currents could be used to provide restrictive criteria to build computational models that more closely mimic physiological conditions. Gating pores have also been studied to better understand the environment (hydrophilic and hydrophobic) and functioning (sequence of activation of the various domains, movement of the S4 segment) of VGICs.

To date, four phenotypes have been clearly associated with the appearance of gating pore currents (HypoPP, NormoPP, arrhythmic DCM and PNH). These pathologies are caused by mutations on Na_v_, Ca_v_ or K_v_ channels. However, suitable environments for the creation of gating pores can be found in most proteins containing a VSD. It is thus surprising that gating pore currents have only been detected in one pathology associated with K_v_ channel mutations. This may be due to the relative novelty of gating pores or to the highly deleterious impact of gating pore currents induced by the tetrameric nature of K_v_ channels. Similar hypothesis might explain why only few mutations located on the S2 or S3 segments of VSDs have been related to the development pathological phenotypes. Such mutations in the GCTC would also disrupt interactions between the GCTC and the S4 segment leading to the appearance of a gating pore current. The resulting gating pore would be constitutively activated as the interactions between the GCTC and the S4 segment would be disrupted for all the possible states of the VSD. Nevertheless, this phenomenon has never been observed and remains to be elucidated.

Future discoveries might reveal pathologies caused by the disappearance of gating pore currents. Indeed mutations that abolish or modify the currents carried by H_v_1 channels might lead to the discovery of an association with various pathologies given their broad distribution.

Lastly, gating pore currents are a novelty in the field of VGICs and are a promising tool for structure-function investigations. Studies on gating pore currents provide new insights into familial diseases and may help identify the causes of various pathologies and, as such, the development of new therapeutic approaches.

### Conflict of interest statement

The authors declare that the research was conducted in the absence of any commercial or financial relationships that could be construed as a potential conflict of interest.
